# Rhythm Processing Across Development: Origins, Links to Language Processing, and Perspectives for Intervention

**DOI:** 10.1111/nyas.70161

**Published:** 2025-12-16

**Authors:** Barbara Tillmann, Usha Goswami, Sahar Moghimi

**Affiliations:** ^1^ Université Bourgogne Europe, CNRS, LEAD UMR5022 Dijon France; ^2^ Centre for Neuroscience in Education, Department of Psychology University of Cambridge Cambridge UK; ^3^ GRAMFC, Inserm UMR1105, Université de Picardie Jules Verne Amiens France

**Keywords:** auditory processing, infancy, music, neurodevelopmental disorders, predictions, speech, temporal sequencing

## Abstract

A wealth of research has investigated rhythm processing in music and speech, revealing shared cognitive and neural correlates and potential transfer effects, as evidenced by shared benefits and shared processing difficulties, as well as effects of stimulation and training programs. In this review article, we first discuss the empirical evidence of rhythm processing in adults and children and highlight the need to extend this investigation to early infancy. We next summarize new experimental evidence of rhythm processing in early infancy, with a focus on prematurely born infants who provide a model of early neurodevelopment. Finally, we present two longitudinal studies as concrete examples for investigating rhythm processing in healthy full‐term infants for nonverbal and speech materials and its tracking over development (here up to 5 years). Altogether, this review aims to motivate new research investigating interindividual differences in rhythm processing in early infancy, along with implications for typical and atypical developmental contexts and potential diagnostic value. It provides evidence for the potential benefit of early rhythm‐based training interventions, which may decrease the cascading effects of early atypical rhythm processing during development.

## 1 Introduction

**FIGURE 1:**
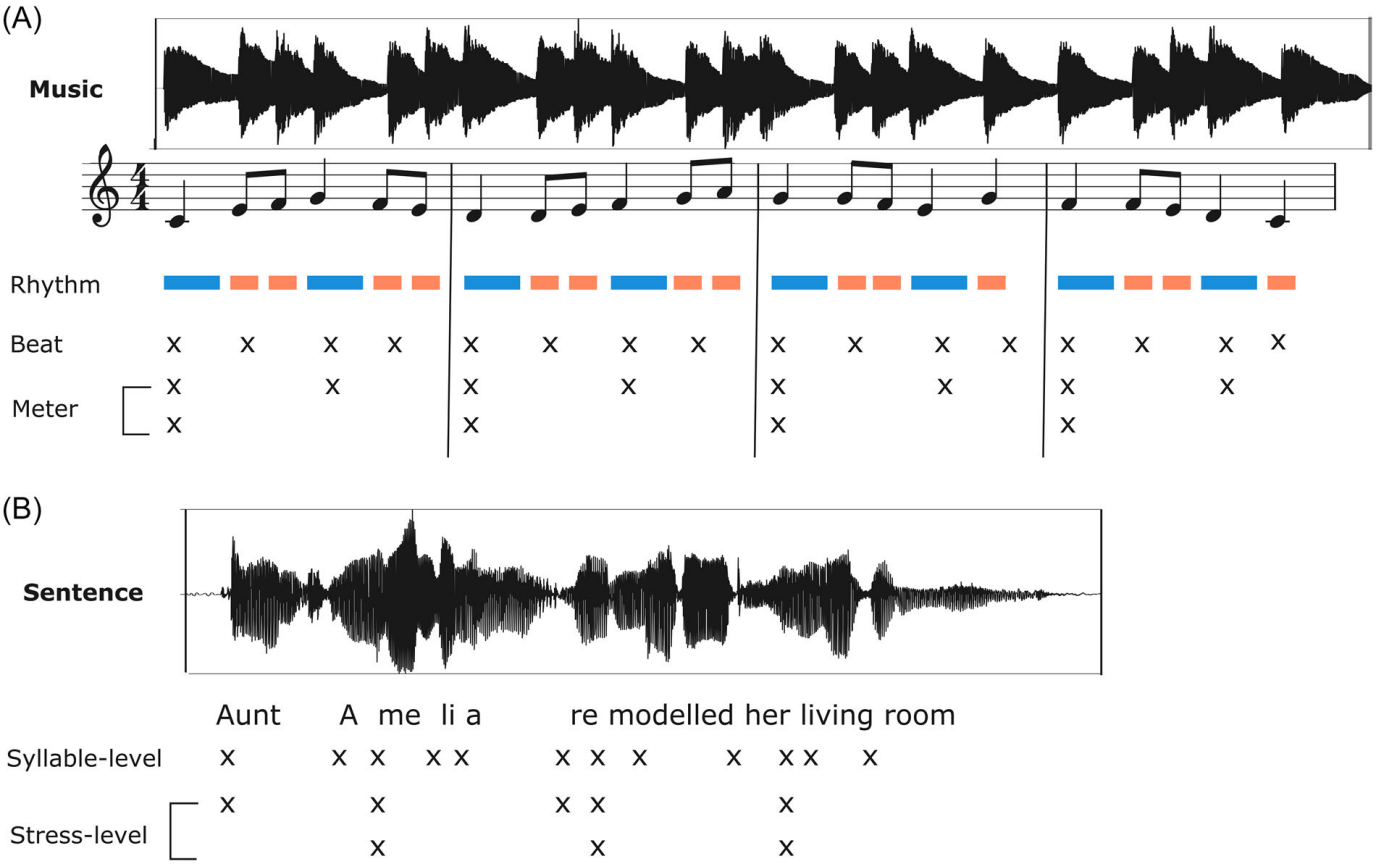
Representations of music (a melody) and speech (a sentence) showing the acoustic waveforms, the melody or sentence represented within the waveform, and the hierarchical temporal structures. (A) The durations of each note are outlined in the rhythm row, the perceived beat is marked with an x in the beat row, and the higher‐level metric structure of the melody is marked with x's in the following two rows. (B) The onset‐time of each syllable is marked on the syllable‐level row, and the higher‐level structure of stressed syllables are marked on the following rows. For the role of amplitude rise times specifically, see Section 4, focusing on infant‐ and child‐directed speech. Reprinted from Figure 1 of Ref. [13]. Reproduced with permission from American Psychological Association. No further reproduction or distribution is permitted.

Music and language share a set of acoustic and structural similarities, including acoustic features, such as pitch and duration differences, and structural features, like syntactic structures and hierarchical organizations (e.g., Refs. [1, 2]). One central characteristic shared by music and language that has attracted particular attention is rhythm. Both music and speech are acoustic signals that are delivered in time and contain temporal structures and regularities related to the duration and timing of events. Rhythm can be defined as temporal patterns created by the onsets and durations of events in a sequence (e.g., Refs. [3, 4]); a definition that applies both to music and speech. In its simplest form, the temporal distance between the onsets is the same between the events, thus leading to an isochronous sequence. However, most rhythmic patterns are nonisochronous, such as long‐short‐short, for example. Acoustic cues are used in both music and speech materials to organize temporal information and also to form hierarchical structures (Figure 1). In addition to cues of loudness, pitch, and timbre, amplitude “rise times” (related to timbre), which relate to the attack time of a sound, are important to rhythm perception (see Section 4).

Most musical rhythmic patterns enable the extraction of an underlying beat or pulse, which is an isochronous sequence marking the time points where listeners might naturally clap their hands. Superimposed over this isochronous pulse, a regular succession of strong and weak beats might emerge, which is referred to as meter or metrical structure, thus leading to hierarchical temporal structures (Figure 1). Importantly, pulse and meter do not need to be implemented in the acoustic realization of the musical material, but can be inferred by the listener, and they thus can be a cognitive construct. While the temporal regularities in music are around isochrony with the pulse/beat level and its metrical structures, this is not the case in adult conversational speech. Infant‐ and child‐directed speech have more isochronous rhythms (see Section 4). Rhythms in adult‐directed speech have been described as “quasi‐regular,” building on patterns of prominence, grouping, and lexical stress (Figure 1). Interestingly, when reading aloud, adults’ spoken renderings are more temporally regular than during adult‐directed conversational speech [5].

**FIGURE 2:**
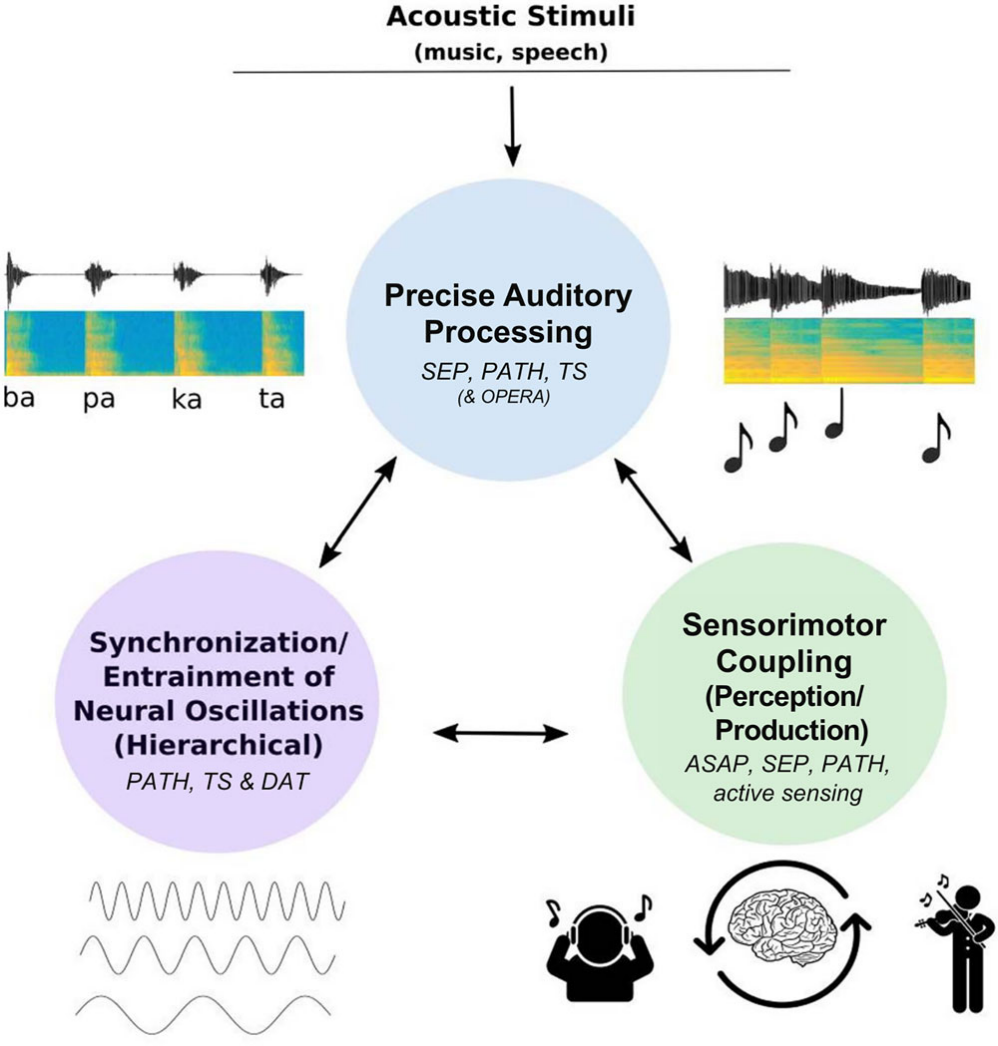
The *processing rhythm in speech and music* (PRISM) framework proposes three common underlying mechanisms for rhythm processing in music and speech: precise auditory processing; synchronization/entrainment of neural oscillations to external rhythmic stimuli; and sensorimotor coupling. These three main mechanisms emerged from previously proposed theoretical frameworks, such as more general frameworks like the Dynamic Attending Theory (DAT [6, 9, 222]) and the OPERA hypothesis (e.g., Ref. [217]) as well as sensory‐motor theories (e.g., Action Simulation for Auditory Prediction, ASAP [143], active sensing [223, 224]) and more specific frameworks that focus on temporal processing, such as the Precise Auditory Timing Hypothesis (PATH [225]), the sound envelope processing and synchronization and entrainment to pulse (SEP) hypothesis [226] and the Temporal Sampling (TS) theory [160] (see Section 4). Adapted from Figure 2 of Ref. [13]. Reproduced with permission from American Psychological Association. No further reproduction or distribution is permitted.

For both music and speech, the temporal regularities implemented in the material allow for developing temporal expectations and guide the perceiver's attention over time for more efficient processing [6, 7, 8, 9]. This process is described by the dynamic attending theory (DAT), which proposes that attention is not evenly distributed over time, but develops cyclically, with internal (neural) oscillators entraining to the external regularities and directing attention over time [6, 8, 9, 10]. The internal oscillators support temporal predictions, facilitate processing, and support segmentation as well as structural and temporal integration. Originally developed for music, the dynamic attending theory has also been applied for language processing [11, 12]. On the basis of the DAT and other previous theoretical frameworks describing partly overlapping mechanisms underlying music and speech processing, Fiveash et al. proposed the Processing Rhythm in Speech and Music (PRISM) framework [13] (see Figure 2). This framework outlines three main mechanisms that appear to be shared across rhythm processing in music and speech/language: (1) precise auditory processing; (2) synchronization/entrainment of neural oscillations to external stimuli; and (3) sensorimotor coupling. Outlining these three mechanisms underlying rhythm processing in music and speech contributes to target the investigation of its cognitive and neural correlates, not only in typical development (in adults and children), but also atypical development, for example, regarding speech and language pathologies. PRISM also provides a framework for investigating the potential benefits of music and rhythmic training or rhythmic stimulation on speech/language processing at the causal level, with perspectives for clinical application.

The present paper focuses on rhythm processing across development and its potential importance for children's cognitive development, in particular for language processing. The goal is to draw out implications for typical and atypical developmental contexts as well as provide perspectives for potential diagnostic and training interventions. Section 2 starts with an overview of research investigating rhythm processing in music and speech for both typical and atypical populations, including cross‐domain transfer effects and rehabilitation perspectives. Most relevant research has assessed adult and child populations using both behavioral and neural correlates. Section 2 reveals the need to understand rhythm processing very early in development and track its progression over time, including its potential transfer to the development of other cognitive capacities. Section 3 thus focuses on current research investigating the neural coding of temporal information starting with the third trimester of gestation, notably in the premature infant's brain, and with some comparison to full‐term born infants. Section 4 presents longitudinal data on rhythm processing development from infancy to toddlerhood, illustrating how measuring rhythm processing early in life can provide insights into later cognitive abilities. These longitudinal studies were based on the Temporal Sampling theory, a sensory‐neural theory proposing a key role for rhythm processing in language acquisition. The paper ends with a call for new research directions investigating interindividual differences in rhythm processing in early infancy as well as its potential diagnostic and therapeutic value.

## 2 Rhythm Processing: From Adults, to Children, to Infants

The similarities and differences in acoustic cues, temporal regularities, and hierarchical structures between music and speech have motivated the combined and/or comparative investigation of rhythm processing in both materials. The link between rhythm processing in music and language has been shown both for competences (e.g., with musical training) and impairments (e.g., in developmental language disorders): increased (decreased) rhythm abilities when processing musical/music‐like material correlated positively (negatively) with language test performance (e.g., grammar). These findings, which have been partly shown also with school‐age children (see below), have led to testing directly for transfer effects with stimulation and training approaches (e.g., Refs. [13, 14]). These more recent developments are building on a rich empirical basis investigating behavioral and neural correlates of rhythm processing in music and language for adults (see also Ref. [15] for new findings related to shared genetic bases). For musical materials, numerous research has studied rhythm perception and production in adults, with a long tradition in music cognition research with behavioral methods (e.g., Refs. [3, 16, 17]) and the investigation of its neural correlates (e.g., Refs. [18, 19, 20]). Similarly, for language materials, the processing of its rhythmic and metric features has been investigated with behavioral measures (e.g., Refs. [11, 21]) and also for its underlying neural correlates (e.g., Refs. [22, 23, 24]), including its influence on syntax and semantic processing (e.g., Refs. [25, 26, 27, 28]). The link between rhythm processing in music and language has been shown with correlational studies (e.g., Refs. [29, 30, 31, 32]; see also Ref. [33]) and the stimulative effect of rhythmic regularities in music‐like materials on subsequent language processing (e.g., Refs. [34, 35, 36, 37, 38, 39, 40, 41, 42]). The potential mediation by common neural resources was further supported by a neuroimaging meta‐analysis identifying shared neural resources of musical rhythm and linguistic syntax processing [43].

This research investigating adults has been extended to children, studying how they process rhythmic features in both music and language materials. Behavioral and neural research has investigated the potential link and overlap between these domains, using correlational and longitudinal approaches, including training interventions. Most studies have targeted children at elementary school age (e.g., from 6 to 10 years old) and preschool age (e.g., from 4 to 6 years old), but some studies also include younger children (e.g., infant/toddler day care) or extend over to adolescence and young adulthood. Correlational studies have shown that behavioral performance in nonverbal, musical tasks testing rhythm perception and production, rhythm discrimination and/or beat synchronization, correlates or allows predicting participants’ performance in various language tasks, such as phonological processing (e.g., Refs. [44, 45, 46, 47, 48, 49]), grammar processing, whether expressive grammar (e.g., Refs. [50, 51, 52]) or receptive grammar (e.g., Refs. [53, 54]), as well as reading and spelling (e.g., Refs. [55, 56, 57]). These cross‐sectional studies have been recently complemented by longitudinal studies following typically developing children over 2 or 3 years (without intervention). Children's beat synchronization performance when they were 6 years old (grade 1) predicted their reading performance at ages 7−9 (Grades 2−5) [58, 59]. Similarly, tapping performance in a sensorimotor‐synchronization task in 7‐year‐olds predicted reading and spelling performance 2 years later [60].

These findings suggesting shared competences across domains have been complemented by studies showing shared impairments across domains, notably in neurodevelopmental language disorders. Children's performance in nonverbal, musical tasks investigating rhythm and beat perception as well as production (e.g., tapping to a metronome or a song) correlated and/or enabled the prediction of their performance in various language tasks, such as tasks targeting phonological processing (e.g., rime awareness, phonological awareness) and reading or spelling performance. This link across domains has been observed for children with dyslexia (e.g., Refs. [61, 62, 63, 64, 65]) and for children with developmental language disorder (previously referred to as speech and language impairment/specific language impairment) (e.g., Refs. [66, 67]). Research in dyslexia (both for children and adults) has further revealed atypical neural tracking of temporal regularity in verbal and tonal materials, as reflected by, for example, atypical neural responses to speech edges and/or altered oscillations in delta or beta frequency (e.g., Refs. [68, 69, 70, 71, 72, 73, 74]). This atypical neural tracking informs for potentially altered predictive timing and sequence entrainment in dyslexia. Indeed, previous reports for typically developing adults had shown that when listening to an isochronous sequence, the power fluctuations of neural oscillations in the beta band (15–25 Hz), both in auditory and motor cortex, may reflect listener's entrainment to the sequence and relate to predictive timing and additional processing (e.g., Refs. [75, 76]).

The observed shared competences and reduced capacities across domains have motivated the testing of training and stimulation programs (e.g., Refs. [77, 78, 79]). For example, proposing 30 weeks of musical/rhythmic activities to a group of dyslexic children led to enhanced phonological awareness and reading performance—in comparison to the same duration of painting activities proposed to a control group of dyslexic children [77]. For typically developing children (7‐ to 8‐years old), 8 months of music training, which focused on rhythmic processing (both perception and production), enhanced word reading accuracy (in comparison to an active control group doing visual art activities and a passive control group) [80]. In addition to these rather long‐term training programs, the benefit of music processing with its regular temporal structures has also been shown in the short‐term with auditory rhythmic stimulation (see Refs. [41, 81] for reviews). In rhythmic cueing studies, a short rhythmic cue that matches one‐to‐one with the accents of a subsequent sentence (i.e., the stressed syllables) enhances phoneme detection compared to an irregular, mismatching cue [82, 83]. In rhythmic priming studies, a regular rhythmic musical prime is followed by a set of naturally spoken sentences. Participants judge the grammaticality of these sentences and succeed better after the regular rhythmic primes in comparison to various baseline conditions (irregular rhythmic primes, environmental sound scenes, contemporary music without beat, silence). This rhythmic priming effect (with its noninformative rhythmic prime) seems to be specific to tasks that require temporal processing and cognitive sequencing (e.g., syntax processing, sentence repetition, syllable segmentation), as it was not observed for visuospatial or linguistic control tasks (e.g., Refs. [84, 85, 86, 87]). A rhythmic priming effect has been shown not only for typically developing children [86, 88] and adults [89, 90, 91], but also for children with developmental language disorders [42, 85, 87, 92] as well as children [42] and adults with dyslexia (including for the P600, reflecting ungrammaticality detection [90]). These training and stimulation data integrate more generally into other findings showing benefits of processing thanks to regular temporal structures, notably on perception and cognition, learning and memory, as well as social interaction (see Ref. [93] for a recent review and the integration of rhythm‐related reward). The temporal regularities can be either directly implemented in the material (as in an isochronous sequence, for example) or extracted by the listener, such as for the beat/pulse or the underlying metrical hierarchy.

Temporal processing difficulties have been reported not only in neurodevelopmental language disorders (in particular, dyslexia and developmental language disorder; e.g., Ref. [94]), but also stuttering, autism spectrum disorder, developmental coordination disorder, and attention deficit hyperactivity disorder (e.g., Refs. [13, 95]). These observed difficulties, together with the empirical data showing shared competences for rhythm processing in music and language as well as the cross‐domain effects of training and stimulation programs, have motivated the Atypical Rhythm Risk Hypothesis [96]. The hypothesis is that atypical rhythm processing early in life might represent a risk for cascade effects of this atypical processing on the development of other cognitive processing abilities, including language processing. Atypical rhythm is defined as impairments in rhythm, beat, and meter processing and synchronization or entrainment, which might affect temporal predictions more generally and thus learning of various features and abilities over development. From this hypothesis follows the need to further investigate the abilities to process rhythm in nonverbal, musical material early in life, aiming to better understand rhythm processing development as well as the potential consequences of atypical rhythm processing on infant development and language processing capacities in particular. This investigation could be structured around the three main mechanisms summarized in the PRISM framework, notably focusing on fine‐grained auditory processing, brain oscillatory networks, and sensorimotor coupling [95, 96]. More targeted investigations of causal mechanisms might allow further understanding of the potential patterns of impairments in pathology (with their different configurations depending on the pathology) and also provide grounds for developing training and rehabilitation approaches, in particular early in life (see Ref. [14]).

The Atypical Rhythm Risk Hypothesis also motivates investigating the very early capacities for rhythm processing, with the aim of detecting early deviation and impairments, as well as the potential benefits of early training interventions in vulnerable populations, with the aim of reducing the detrimental cascade effects of atypical rhythm processing and helping children reach developmental milestones on time. In this line, Section 3 presents research investigating rhythm processing abilities in very early infancy, notably before the age of term (37−41 weeks of gestational age) and in infants who are born prematurely (before 37 weeks of gestational age), with some comparison to full‐term born infants. One of the consequences of premature birth is the infant's deprivation of the experience of rhythmic regularity that a full‐term born infant has in the womb, representing a potential risk factor for the infant's development and representing a potential target population that can benefit from early rhythmic interventions.

## 3 Neural Rhythm Processing: Development Across the Third Trimester of Gestation

Evidence shows that newborns can process the rhythmic hierarchy of auditory sequences at and near term [97, 98, 99, 100, 101]. Behavioral studies (e.g., preferential looking or pacifier sucking paradigms) have shown that newborns can discriminate languages from one another based on their distinct rhythms [102, 103, 104]. Furthermore, neural data indicate that the newborn's brain follows the speech envelope [105, 106, 107]. These observations raise important questions about the prenatal development of auditory rhythm processing, notably as auditory rhythm experience begins remarkably early in human life. The unborn child is exposed to different rhythmic sounds, from the omnipresent maternal rhythms to the nuanced patterns of maternal and external rhythms of speech and music transmitted to the fetus. Growing evidence suggests that this early exposure is not merely incidental, but experience‐expectant; it plays a fundamental role in early development. The auditory system becomes functional during the late second trimester of gestation [108], and the thalamic afferents arrive at the cortical plate around the beginning of the third trimester of gestation [109]. This period represents a critical checkpoint along the neurodevelopmental journey during which exogenous stimuli—alongside genetic events and spontaneous neural activity [110, 111]—begin to significantly contribute to neuroplastic changes underlying the development of sensory systems and neural networks (see Ref. [112] for a review). Although there is evidence for reorganization and compensation in neural circuits (see Ref. [113] for a review), experience‐dependent refinement of circuits and functions [114] underscores the importance of environmental factors during this phase of high plasticity. Neurodevelopmental phenomena and milestones determine whether and how sensory information is encoded. Development of sensorimotor networks precedes that of higher‐order integrative networks, where the processes occurring from neurulation until complete circuit refinement are considered to expand over a longer time window into adulthood than those in sensory or motor cortical regions [115]. In addition, early development of local wiring and long‐range cortical connections is followed by a reorganization of circuits toward mature cortical functional networks with multiple modes of efficient information encoding and communication. At the same time, rapid oscillations emerge over subcortical and cortical networks with changes in the inhibition‐excitation balance, and communication is formed on different oscillatory regimes, continuing into adulthood [111, 116, 117]. All these mechanisms, in addition to their possible delay or deviation associated with disorders, can impact how and what aspects of environmental information are encoded.

Neuroimaging techniques allow studying the neural responses to temporal and rhythmic information before the emergence of behavioral responses, therefore, making it possible to investigate the early neural capacities and their evolution with development, despite the absence of synchronized limb motor activity. Cortical auditory responses have been recorded as early as 27 weeks gestational age in the fetus, using magnetoencephalography [118], and in premature newborns using electroencephalography (EEG) and functional near‐infrared spectroscopy [119, 120]. While fetal magnetoencephalography can allow the study of neural responses in the fetus' “natural” environment, characterizing these responses and uncovering the underlying mechanisms remain difficult due to high physiological and environmental magnetic fields, resulting in a low signal‐to‐noise ratio. This makes it challenging to draw conclusions about the development of neural capacities. Although extrauterine development of premature newborns does not perfectly reflect prenatal neurodevelopment [121, 122, 123], studying premature newborns without severe injuries in the days immediately following birth can provide valuable insights into how rhythm is encoded at different gestational ages. In this context, EEG enables investigation of the neural mechanisms underlying early cortical responses, while functional near‐infrared spectroscopy can reveal the activation of specific cortical regions in response to stimulation.

A new EEG project based at the University of Picardie, the PreMusic project,^1^ will investigate the development of auditory rhythm perception during the third trimester of gestation, starting from 28 weeks gestational age, which is approximately when the thalamic afferents arrive at the cortical plate. The project examines the impact of early rhythm‐based musical interventions on the neural capacity of premature newborns to encode auditory rhythms. In the context of this project, tracking brain responses in EEG to periodic stimuli in sleeping newborns has already revealed that before the age of term, the premature brain between 32 and 34 weeks gestational age follows the nonisochronous rhythmic structure [124, 125] of auditory sequences at different periodicities [126]. The premature brain even shows evidence for predictive beat timing. When presented with a 6‐beat rhythm composed of “tone‐rest‐tone‐tone‐tone‐rest” [127], periodic modulation (increase and decrease) of oscillatory power in the frequency range 7−12 Hz aligned to the isochronous beat with peak timing of oscillatory power preceding the beat event [128]. Importantly, this study indicates that the premature brain encodes an isochronous beat when presented with a nonisochronous rhythm, suggesting that the observed beat‐based power modulation may be related to intrinsic predictive processes of beat timing [129, 130].

These neural capacities develop progressively during the third trimester of gestation. Evidence for this comes from findings suggesting that neural tracking of different tempi (different periodic cycle durations) in the aforementioned 6‐beat rhythm, quantified by a stimulus‐brain synchronization index, appears to emerge at different times between 28 and 37 weeks gestational age [131]. The rhythmic structure of the auditory sequence used in this study led to a beat frequency of 3.33 Hz and two meter‐related frequencies of 1.67 and 1.11 Hz. While slow periodicities (1 and 1.67 Hz) began to be encoded near the age of term, faster periodicity (3.33 Hz) was already encoded at the beginning of the third trimester of gestation. The underlying maturational neural mechanisms of this observation are yet to be investigated. Neural models based on oscillator‐ and predictive‐coding theories that are adjusted to neurodevelopmental evidence during this period can provide a valuable opportunity to address how neurodevelopment can shape the neural response to different periodicities at different timescales and the rhythmic hierarchy that is shared between speech and music [132, 133].

Growing evidence shows the capacities of the premature brain for processing auditory stimuli [134, 135]. These neural responses are not necessarily from “*low‐level*” processing in the auditory networks. It seems that a transient nexus can provide a distant long‐range connection at the very early stages of neurodevelopment that is later replaced by more stable cortical networks with progressive neurodevelopment [136]. The hypothesis that external stimuli, and in the context of this paper, external rhythmic inputs, are already being processed in distributed networks makes sense from the developmental point of view. Considering the plasticity of networks involved during later developmental stages in rhythm processing in relation, for example, with language processing, social interaction cues, motor and musical behavior, early long‐range connections distributed across the cortex are highly likely to be involved. Nevertheless, the cortical structures underlying very early rhythm processing are still unknown.

Neuroimaging evidence in human adults [137, 138, 139, 140, 141, 142] and theoretical frameworks, such as the Action Simulation for Auditory Prediction hypothesis [143], suggest that musical and speech rhythm perception elicit activity across cortical premotor and sensorimotor areas, even when no overt movement is involved. It is yet not clear whether the early neural capacity to track rhythmic progressions relies solely on basic “low‐level” encoding in auditory networks or whether it involves distributed cortical/neural structures, including sensorimotor regions. In an effort to address this question, the PreMusic project investigated cortical hemodynamic activity in premature infants using functional near‐infrared spectroscopy while exposing them to auditory rhythmic sequences. The findings revealed that exposure to nonisochronous rhythms with a stable beat elicits enhanced activity in the cortical regions of the auditory pathway, extending dorsally beyond the auditory cortex and into the sensorimotor regions [144]. However, a significant limitation of this study is that the near‐infrared spectroscopy optodes did not fully cover the sensorimotor regions, requiring caution in interpreting these results and needing refinement with better coverage of premotor/sensorimotor regions to confirm this observation. That the sensorimotor and premotor regions may exhibit increased activation in response to a salient beat in premature infants, combined with prior evidence of predictive beat processing at this stage of neurodevelopment [128], supports the hypothesis of an immature, yet emerging, beat‐based predictive timing cortical network. Although the brains of premature and full‐term newborns already exhibit responsiveness to auditory rhythms and show evidence for neural tracking of beat and meter in rhythmic input, precise neural encoding of the nested periodicities present in musical rhythm and speech prosody cannot yet be expected. The capacity to encode the hierarchical structure of rapidly unfolding sequences and to extract nested temporal regularities requires communication at different timescales in distributed subcortico‐cortical circuits of long‐range synaptic connection. These capacities undergo substantial and elaborative development during infancy, shaped by interactions with the environment and early experiences [145, 146, 147, 148, 149, 150], continuing to refine into adulthood.

Put together, the studies reviewed here highlight the premature brain's early capacity to process auditory rhythm. As reviewed in Section 2, many neurodevelopmental disorders are associated with impaired time and rhythm processing. As will be reviewed in Section 4, neural encoding of the rhythmic regularities in infant‐directed speech has revealed links between early neural encoding of rhythmic information and later language development. These findings support the hypothesis that impaired rhythm processing might lead to detrimental cascade effects over development. Evidence discussed in the present section reveals the importance of early sensory experience in the development and refinement of neural circuits during brain development (see also Refs. [111, 151, 152, 153] for reviews in the visual domain and [154, 155] in the auditory domain). Premature newborns—especially those born extremely preterm—are deprived of the fetal auditory environment and instead exposed to the atypical acoustic landscape of the neonatal intensive care unit. Preterm birth is associated with an increased risk of neurodevelopmental disorders [156, 157], with susceptibility rising as gestational age at birth decreases. Alongside pathologies that are associated with premature birth, early deprivation of sensory and, more specifically, auditory experience can impact early neurodevelopment. Recent research suggests that early interventions can influence the resting‐state functional neural networks expanding over frontal, auditory, and sensorimotor regions of the developing brain [158, 159]. However, the potential impact of preterm early musical, and specifically rhythm‐focused interventions on the neural encoding of rhythm in both music and speech remains unexplored. Given the high plasticity of the brain at this stage, early rhythm‐oriented musical interventions may improve initial wiring, yielding both short‐term and long‐term benefits for rhythm processing, with potential transfer to the early development of other cognitive capacities. This hypothesis requires validation through data that illustrate the maturation trajectories of neural rhythm processing both in and ex utero, as well as the impact of early interventions.

## 4 Rhythm Processing, Hierarchical Acoustic Structures, and Language Acquisition: Longitudinal Infant Studies Based on Temporal Sampling Theory

The research reviewed in Sections 2 and 3 suggests that impaired rhythm processing might lead to a detrimental cascade of effects over development. One way of investigating this empirically is to use the lens of Temporal Sampling theory, which proposes that individual differences in sensitivity to rhythm structures may be related to individual differences in language acquisition [160]. Temporal Sampling theory was proposed initially to explain why impaired rhythm processing may relate to impaired linguistic (phonological) processing in developmental dyslexia [64], and was then extended to language acquisition following a series of speech modeling studies [161, 162]. In brief, Temporal Sampling theory adopts an acoustic perspective on rhythm based on the “amplitude envelope” of speech and on the discrimination of amplitude “rise times.” The speech amplitude envelope describes the shape of the overall sound wave reaching the human ear. Amplitude “rise times” are rates of transition from lower to higher amplitude (loudness) that co‐occur at multiple rhythmic time frames within speech. When we speak, we are creating sound waves (perceived as loudness fluctuations) which the auditory system encodes as amplitude modulations (AMs). The brain tracks the temporal structure of these AMs, and low‐frequency AMs < 10 Hz dominate the speech envelope. It is these low‐frequency AMs that are thought to govern the computation of speech rhythm patterns [163].

The brain picks up all the AM changes in the envelope, which vary naturally in both pitch and loudness because of the way that speech articulators work. The brain then automatically aligns its own intrinsic rhythms (“brain waves” or neural oscillations) to these sound rhythms. Accurate cortical tracking of the speech envelope is triggered by the automatic phase‐resetting of endogenous brain rhythms (oscillations in electrical signaling) by acoustic “edges” in the speech signal (amplitude rise times) that usually signify syllables [164, 165]. The neural oscillators also form a mechanistic hierarchy via inherent oscillatory coupling. Networks of neurons are dynamically modulated from slower to faster bands in a top‐down manner, with the delta (0.5–4 Hz neural oscillatory band) at the top. Delta phase modulates theta phase, delta phase modulates beta amplitude (12−30 Hz), and theta phase modulates gamma amplitude (a faster band, >30 Hz [166]).

**FIGURE 3:**
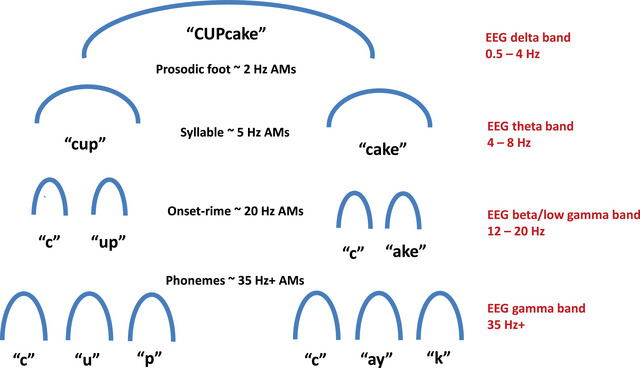
Temporal Sampling theory and the Amplitude Modulation Phase Hierarchy. Schematic depiction of the nested AM information that supports linguistic parsing of phonological units, with the matching brain rhythms. By modeling each AM cycle as one phonological unit, automatic alignment of neuronal oscillatory networks at matching temporal rates to the AM hierarchy enables accurate encoding and parsing of the speech signal. This speech‐brain alignment appears to be “out of time” for children with language disorders. Reproduced with permission from Ref. [94].

As noted previously, the newborn infant brain can track the speech envelope [105]. However, languages like English and German are assumed by linguists to have different rhythm patterning from languages like French and Spanish (stress‐timed vs. syllable‐timed rhythms). New speech modeling adopting an AM‐focus has shown that there is a systematic and matching AM structure nested inside the speech envelope for all of these languages (Spectral‐Amplitude Modulation Phase Hierarchy models, see Refs. [5, 161]). Temporal Sampling theory suggests that this AM information at slower temporal rates may be one basis of language acquisition (bands of AM <10 Hz) [162]. Speech modeling studies based on Spectral‐Amplitude Modulation Phase Hierarchy models have shown that when infant‐ and child‐directed speech (or metrical poetry) are analyzed from an AM perspective, three core AM bandings are reliably found centered on ∼2, ∼4/5, and ∼20 Hz [161]. These bandings correspond to the timing of neural oscillations in the delta, theta, and beta/low gamma frequency ranges, and one temporal cycle at each rate also corresponds to phonological units in the linguistic hierarchy (see Figure 3). These three core AM bandings are not characteristic of quasi‐rhythmic sounds found in nature, such as birdsong, rain, and wind. However, the same three core AM bandings characterize different human musical genres, such as rock, classical music, and jazz [167]. Accordingly, when infant‐ and child‐directed speech are modeled, the AM‐structure of music and speech is highly similar [167].

**FIGURE 4:**
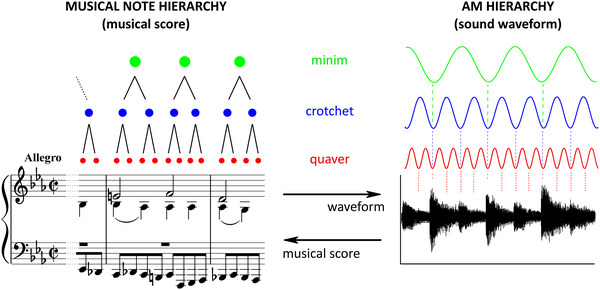
Temporal Sampling theory and musical note hierarchies. The left‐hand panels depict rhythmic representation by musical score and beat structures, and the right‐hand panels depict the corresponding sound waveform and its AM decomposition. The music is a part of the 33 Variations on a waltz by Anton Diabelli, Op. 120 by Ludwig van Beethoven. The musical rhythm structure is hierarchically organized based on note values (left), which match rates of amplitude modulation (AM, right). In the framework of Temporal Sampling theory, the AM bands (right) equate temporally to neural oscillatory rhythms at rates of ∼2 Hz (delta band), ∼4 Hz (theta band), and ∼20 Hz (beta/low gamma band). Reproduced with permission from Ref. [227].

The hierarchical acoustic AM‐driven rhythm relationships that characterize both human music (see Figure 4) and infant‐/child‐directed speech depend on systematic *phase relations* (rhythmic synchronicity) between the bands of AMs, as revealed by the computational modeling [161, 167]. For both speech and music, the phase relationships between the two slower AM bandings centered on ∼2 and ∼4/5 Hz are of particular importance for identifying rhythm patterns [162, 167, 168, 169]. Infant‐directed speech and human music both show a modulation peak in the delta band (∼2 Hz, interestingly adult‐directed speech shows a modulation peak in the theta band, at ∼5 Hz, note that modulation peaks indicate where energy is concentrated in the signal). In addition to the difference in modulation peaks, the strength of the phase relations between the slower ∼2 and ∼4/5 Hz AM bandings is significantly weaker in adult‐directed speech than in infant‐directed speech.

Further, this enhanced ∼2 Hz/∼5 Hz AM phase synchronization found in infant‐directed speech is also the dominant phase synchronization between AM bandings in the amplitude envelope of music [33]. Associated studies in the Temporal Sampling framework have shown that when both AM bands peak together, listeners perceive a strong (stressed) syllable, whereas when a trough in the slower AM band (∼2 Hz) coincides with a peak in the faster AM band (∼5 Hz), listeners perceive a weak syllable [169]. In music, the Spectral‐Amplitude Modulation Phase Hierarchy modeling suggests that the phase alignment of these two AM bands may map to the perception of minims and crotchets (see Ref. [167]; Figure 4). Interestingly, infant lullabies across languages use this 2 Hz rate (120 beats per minute [170]).

Following this theoretical perspective, the two longitudinal infant studies reviewed here took amplitude rise times, the dominance of the ∼2 Hz AM banding, and the importance of ∼2 Hz/5 Hz AM phase relations as key acoustic measures that may predict language outcomes if encoded accurately by the infant brain. The two longitudinal studies are the Cambridge UK BabyRhythm study of neurotypical infants [171], and the Seeds of Literacy (SEEDS) study of Australian infants at family risk for dyslexia [172]. In essence, these two longitudinal infant studies investigated the Temporal Sampling hypothesis that the automatic computation of acoustic rhythm patterns by the brain provides the hidden “glue” for building a spoken language system.

The longitudinal Cambridge UK BabyRhythm study involved 122 infants who were followed from the age of 2 months. The infants visited the lab eight times during their first year, and listened to sung nursery rhymes and two kinds of rhythmic input delivered at 2 Hz, verbal (a speaker repeating the syllable “ta” at a rate of 2 Hz in infant‐directed speech) and nonverbal (a drumbeat at 2 Hz). Brain responses were recorded at 2, 4, 5, 6, 7, 8, 9, and 11 months using EEG while infants listened passively to these different acoustic inputs, and language outcomes were then measured from 12 months on (e.g., receptive and productive vocabulary, nonword repetition [phonology], simple grammar). The Cambridge BabyRhythm data so far, summarized in Table 1, support the idea that speech‐brain alignment to the ∼2 Hz delta‐band AM information is fundamental to language acquisition [171, 173, 174, 175, 176]. Analysis methods using multivariate temporal response functions [177] allowed recreating the heard speech envelopes from the brain responses of the infants in the EEG delta (0.5−4 Hz) and theta (4–8 Hz) bands, which encode the two slowest speech AM bandings. Signal reconstruction enabled “reading out” the aspects of the speech signal that were represented by the early learning brain. The accuracy of speech‐brain alignment for the slowest AM changes (∼2 Hz, EEG delta band) was significantly higher than the accuracy for the slightly faster AM changes (∼5 Hz, EEG theta band [173, 174]) at all ages measured (4, 7, and 11 months). Only delta‐band speech‐brain alignment was a significant predictor of later language outcomes, with greater accuracy of neural alignment in the delta band at 11 months predicting better vocabulary and better phonology at age 2 years [174].

**TABLE 1:** Neural Predictors of Language Outcomes in the Cambridge BabyRhythm Study (*N* = 113) using the 11 months estimate (multiple Temporal Response Function analysis) of low‐frequency cortical tracking of nursery rhymes sung to a 2Hz beat, the 4 months estimate of phase‐amplitude coupling in response to the nursery rhymes, and the preferred AV phase measures to rhythmic 2Hz repetition of syllables vs drumbeats at any month of ages 2 – 9 months. Please note that more nuanced information is available in the BabyRhythm publications [173, 174, 175, 176].

Neural Measure	Vocab 18m (CCT)	Vocab 24m (CDI)	Phonology 24m
Delta band cortical tracking	NS	**SIG**	0.09
Theta band cortical tracking	NS	NS	NS
Delta‐gamma phase amplitude coupling	NS	**SIG**	NS
Theta‐gamma phase amplitude coupling	**SIG**	**SIG**	**SIG**
Delta power	NS	NS	NS
Theta power	NS	**SIG** negative	**SIG** negative
Theta/delta ratio	NS	**SIG** negative	0.07 negative
Preferred AV phase in delta, syllable	**SIG**	**SIG**	NS
Preferred AV phase in delta, drumbeat	NS	**SIG**	SIG
Preferred V phase in delta, syllable, 8 months	NS	**SIG**	NS

*Note*: Vocab [vocabulary] measures = CDI [Communicative Development Inventory, parent‐led] comprehension, CDI production, CCT [Computerized Comprehension Task, infant‐led]; Phonol [phonology] measures = accuracy of nonword repetition of items at 3 syllable lengths (e.g. “kish”, “punky”, “tanina”) measured as either syllables correct or consonants correct; **SIG** = <0.05; NS = non‐significant; AV = audio‐visual speech; V = visual‐only speech.

Regarding the simple rhythmic inputs at 2 Hz (“ta” and drumbeat), the phase or timing of infants’ peak neural responses was another significant predictor of their later language outcomes from 2 months onward, for both auditory and visual‐only speech (in the visual‐only speech condition, the infants watched a talking head silently mouthing “ta”) [175, 176]. Put nontechnically, individual differences in how accurately the infant brain was “on the beat” in terms of responding to rhythmic inputs at 2 Hz from 2 to 9 months, both acoustic and visual, predicted receptive and productive vocabulary and nonword repetition. For visual‐only speech, the data suggested that by 8 months, visual tracking affected the *timing* of the auditory neural response, possibly indicating resetting of the auditory cortex to the “optimal” state for processing anticipated succeeding vocalizations [178]. The grammar measures could not be included in these analyses, as too few infants provided grammatical data at age 2 years. Nevertheless, the neural phase data for vocabulary (based on the MacArthur‐Bates Communicative Development Inventories) and phonology (nonword repetition) suggest that the neural *prediction* of speech rhythm information is a critical factor in explaining individual differences in language acquisition.

Another methodological advance using signal processing based on a multivariate temporal response function and that incorporates acoustic features and spectrogram information in addition to the envelope has enabled some of the phonetic information being encoded by the infants’ brains to be revealed from the same EEG data [179]. Di Liberto and colleagues used the same nursery rhyme EEG data as in Ref. [171], but assessed which phonetic features were being encoded by the infants’ developing language systems. It was found that the neural tracking of phonetic features emerged rather slowly across the first year of life. Identifying the phonetic features was far from complete when the brain measurement part of the Cambridge BabyRhythm project ended, at 11 months of age. This provides an interesting contrast with the rhythmic information in speech, which was recorded with high fidelity from the first measurement period (2 months). Phonetic information was still sparse at 11 months, which is the age at which most infants begin to say their first words. This may suggest that the phonological structures that infants use to decode and produce speech are not initially phoneme based.

For typically developing infants and young children, learning the rhythm patterns of their language is automatic and unconscious. The infant brain appears to learn the AM‐driven rhythm patterns described above via automatic speech‐brain alignment, focusing first on global rhythm patterns and then extracting more fine‐grained phonetic information. The second longitudinal infant study, conducted from a Temporal Sampling perspective, SEEDS, followed a smaller cohort of infants (∼50) from 5 months of age. This second longitudinal data set suggests that having poor speech rhythm discrimination early in life makes language acquisition more challenging. In the SEEDS family risk study, infants who were at family risk for dyslexia were worse at discriminating the acoustic cues (amplitude rise times) that trigger automatic speech‐brain alignment than no‐risk infants when aged 10 months [172]. This could suggest impaired neural tracking of the speech envelope, with negative effects for language acquisition, as also predicted by the Atypical Rhythm Risk Hypothesis [96]. Interestingly, children with dyslexia in many languages are also worse at discriminating the amplitude rise time cues that help the brain to “lock on” to the AM‐driven rhythms in speech (e.g., Refs. [180, 181]). In the SEEDS study, those infants who were worse at discriminating amplitude rise times had the poorest vocabulary development at age 3 years [182]. The at‐risk infants also showed slower word learning as toddlers (19 months), poorer overt rhythmic skills as 4‐year‐olds (e.g., tapping in time with a beat), and worse prereading skills as 5‐year‐olds (e.g., letter‐sound knowledge, see Refs. [183, 184, 185, 186]).

Usually, a child's phonological system develops as a natural part of language acquisition, with implicit learning of the incidence and patterning of the different phonological units and speech sounds that make up words facilitated by speech‐brain alignment to the AM hierarchy. Multiple studies show that in developmental dyslexia, the phonological system is impaired at many levels, including the rhythmic level of phrasal prosody, the word level of syllable patterning, and the intraword level of onset‐rime (where syllables are divided at the vowel, as in f‐at, sp‐at, spl‐at). All these levels of phonology are poorly encoded in dyslexia, complicating the alphabetic learning of phonemes (phonemes typically correspond to the sounds made by individual alphabetic letters, s‐p‐l‐a‐t). Children with dyslexia show impaired “phonological awareness” (the ability to recognize and manipulate sounds in spoken words) at all these linguistic levels [187].

The infant studies conducted to test Temporal Sampling theory suggest that the impaired mechanisms contributing to later phonological difficulties are already present in infancy, related to reduced discrimination of amplitude rise times, impaired speech‐brain alignment at slower AM rates, and less accurate phase alignment to the “beat.” However, these infant studies also suggest novel perspectives for remediation. Oral interventions that match motor rhythms to the rhythms of speech, such as tapping or drumming along to strong‐weak syllable patterns while speaking, appear to be quite effective for young children [78], and for infants, these interventions could be adapted to incorporate lap‐bouncing routines in time with the adult singing or speaking rhythmically, or with other rhythmic movements of the infant. Neurally, children with dyslexia show atypically high theta‐delta ratios during natural speech listening [188], and infants with higher theta‐delta ratios in the Cambridge UK BabyRhythm studies had worse phonological outcomes [174]. The theta‐delta ratio during natural speech listening can be improved by filtering speech in a way that amplifies the rise times, to date, this has only been tried with children [189]. Adults with dyslexia can learn to reduce their theta‐delta ratio during natural speech listening when they experience a brain−computer interface that provides visual feedback (a spaceship moving upward or downward). The movements of the spaceship are correlated in real time with a higher versus lower theta‐delta ratio, and adults can learn to keep the spaceship in the position that optimizes this ratio, with beneficial subsequent effects for nonword reading [190].

## 5 Conclusion and Perspectives

The time is ripe for the development of new research directions investigating interindividual differences in rhythm processing, particularly in early infancy, when the most benefits from targeted interventions may be expected to accrue. A focus on this population also enables testing of potential future diagnostic and remedial implications. Many studies reviewed here make the case for the potential benefit of rhythm‐based training interventions, which could decrease the cascading developmental effects of atypical rhythm processing. These research directions also integrate with other research efforts to study auditory processing in infants, notably other studies comparing infants “at risk for dyslexia” with “no‐risk” infants and neonate studies comparing the tracking of temporal regularities in native versus non‐native speech (see Refs. [191, 192, 193, 194, 195, 196, 197, 198, 199, 200]). Atypical tracking of temporal regularities could also be a potential indicator of developing autism spectrum disorder, with autistic toddlers exhibiting more variable rhythmically entrained eye‐looking when viewing infant‐directed singing than do typically developing toddlers [201]. Accordingly, increased research efforts need to be invested in building and validating brief rhythmic processing tests with prognostic value at the individual level, and with the goal of detecting atypical rhythm capacities, including at birth, and measure potential training and stimulation effects. For these tests, EEG measures might be particularly appropriate for testing at an early age, thus as testing sleeping newborns or premature infants (e.g., Refs. [128, 131]), but can be complemented by eye‐movement measurements (e.g., Refs. [202, 203]) or movement measures to music or rhythmic regularities (e.g., Refs. [204, 205, 206]). These can then further be complemented by behavioral rhythm perception and production tasks in toddler and older children (e.g., Refs. [207, 208, 209, 210]).

Longitudinal studies are needed to investigate potential consequences of different degrees of rhythm capacities later in development, and to create appropriate intervention approaches in case of reduced capacities (see also Refs. [13, 96, 211]). We here suggest that this research effort should extend to newborns and also to premature infants. Premature infants unavoidably experience early environmental deprivation of rhythmic regularity, which a full‐term born infant has in the womb. This represents an additional risk factor as well as potentially fertile terrain to implement rhythmic stimulation programs in the neonatal intensive care unit (see Section 3). Promising findings have been recently reported of music exposure in the neonatal intensive care unit, yielding beneficial effects. For example, music exposure appeared to enhance the development of sensory, cognitive, and socioemotional functional neural networks (e.g., Refs. [158, 212]), which are often impaired with prematurity [213, 214]. Future research needs to study in greater depth the potential benefits of early rhythmic stimulation and also investigate whether these beneficial effects remain later in development, for both typically developing infants and at‐risk infants. Understanding whether these benefits persist over time and contribute to improved developmental trajectories in at‐risk populations could have profound implications for early intervention strategies.

The motivation of using music to stimulate rhythm processing and target potential transfer to language and speech processing can further be motivated by a more general framework than PRISM or Temporal Sampling theory: the OPERA framework [215, 216, 217]. This framework rests on the hypothesis that music processing involves neural resources that are shared with other cognitive mechanisms (O for “overlap”) and requests higher precision (P). This is reinforced by the emotional (E) power of music, which results in the engagement of the reward system (e.g., Refs. [218, 219]), crucial for sustained exigent training. Repeated (R) practice of music is combined with enhanced attentional (A) resources during musical training. Even though originally developed for musical training in general, the OPERA framework also applies for rhythm in particular. Rhythm processing in music shares mechanisms with rhythm processing in language, involves high precision and attention, and is involved in the emotional power of music with the engagement of the reward system (e.g., Refs. [220, 221]). In particular, rhythm‐mediated reward might allow for boosting learning and memory as well as social connection and interpersonal synchronization [93], all relevant for infants’ development and potential stimulation and intervention approaches, whether for typically or atypically developing brains [9].

## Author Contributions

Initial draft writing and revisions (B.T., S.M., and U.G.).

## Conflicts of Interest

The authors declare no conflicts of interest.

## Footnotes

1
https://gramfc.u‐picardie.fr/premusic‐babymusic/
